# A bidirectional Mendelian randomization analysis of the causal relationship between immune cells and high-grade squamous intraepithelial lesion

**DOI:** 10.1097/MD.0000000000045695

**Published:** 2025-11-21

**Authors:** Zhenyu Wang, Lu Yang, Chunxiao Li, Ping Jiang, Junjie Wang

**Affiliations:** a Department of Radiation Oncology, Peking University Third Hospital, Beijing, China; b Department of Ultrasound, Peking University Third Hospital, Beijing, China; c Institute of Medical Technology, Peking University Health Science Center, Beijing, China.

**Keywords:** causality, HSIL, immune cells, Mendelian randomization, reverse causality

## Abstract

High-grade squamous intraepithelial lesions (HSIL), critical precursors to cervical cancer, are associated with persistent human papillomavirus infection and immune dysregulation, yet the causal role of specific immune cell phenotypes remains unclear. To investigate bidirectional causal relationships, we performed a 2-sample Mendelian randomization (MR) analysis using European genome-wide association study data: 731 immune cell phenotypes and HSIL. Instrumental variables (*P* < 1 × 10⁻⁵) were selected under MR assumptions, with inverse-variance weighted as the primary method. Forward MR (immune cells to HSIL) identified 67 phenotypes nominally associated (*P* < .05); after FDR correction (FDR < 0.20), IgD on transitional B cells were protective, while increased CD3 on CD39+ Tregs elevated risk. Reverse MR (HSIL to immune cells) indicated HSIL causally reduces naïve CD4− CD8− T cell, HVEM on effector memory CD8+ T cells, FSC-A on lymphocyte and FSC-A on T cells (FDR < 0.20). This study provides genetic evidence for causal roles of specific immune phenotypes in HSIL pathogenesis, suggesting immunotherapeutic targets, while revealing HSIL-induced immune exhaustion patterns.

## 1. Introduction

High-grade squamous intraepithelial lesion (HSIL) is a critical precursor of invasive cervical cancer (ICC) and is strongly associated with persistent human papillomavirus (HPV) infection.^[1]^ While HPV is the primary etiological factor, the progression from HSIL to ICC is a multifactorial process influenced by complex alterations in the tumor immune microenvironment (TIME).^[2]^ Aberrant signal transduction pathways,^[1]^ metabolic reprogramming,^[3]^ and uncontrolled cell proliferation significantly contribute to carcinogenesis. Most importantly, immune evasion mechanisms^[4,5]^ and alterations in the TIME are recognized as key factors in promoting the pathogenesis and progression of HSIL and ICC.

Despite extensive research into the role of HPV infection in cervical carcinogenesis,^[6]^ the specific contributions of immune cells to the pathogenesis and progression of HSIL remain incompletely understood. An abnormal cancer-immunity cycle, which causes weak immune responses, including impaired antigen presentation, altered cytokine profiles, immune cell infiltration and immune cell dysfunction, has been implicated in the pathogenesis of HSIL.^[7]^ These immune alterations may disrupt the immune surveillance of HPV-infected cells, fostering an environment conducive to persistent infection and cellular transformation.

The aim of this study was to investigate the causal relationship between immune cells and the risk of HSIL development via a 2-sample bidirectional Mendelian randomization (MR) approach. By leveraging genetic variants associated with various immune cell populations, we aimed to elucidate the potential causal role of immune responses in the pathogenesis of HSIL. This analysis provides a novel perspective on the underlying mechanisms through which immune cell dysregulation might contribute to the development of HSIL and their progression to ICC.

## 2. Materials and methods

### 2.1. Study design

**Figure 1.**
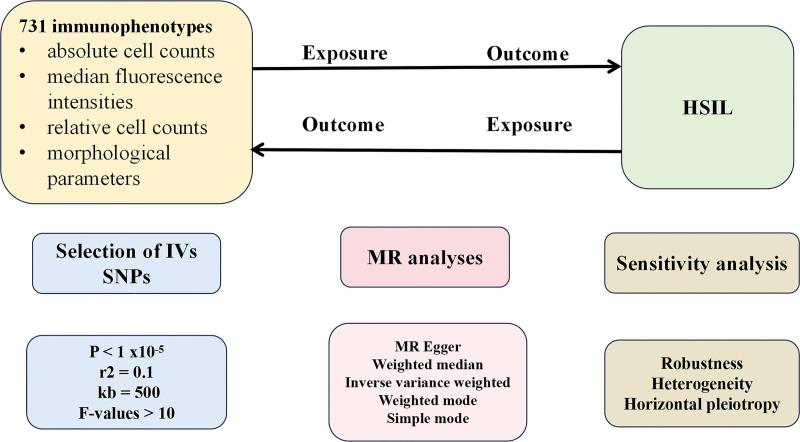
Mendelian randomization flowchart for the analysis of 731 immunophenotypes and HSIL associations. HSIL = high-grade squamous intraepithelial lesion.

Comprehensive bidirectional 2-sample MR analysis was performed to determine the causal associations between immune cell phenotypes and HSIL in this study. The flowchart of the 2-sample bidirectional Mendelian randomization analysis for immune cell phenotypes and the risk of HSIL is depicted in Figure 1.

**Figure 2.**
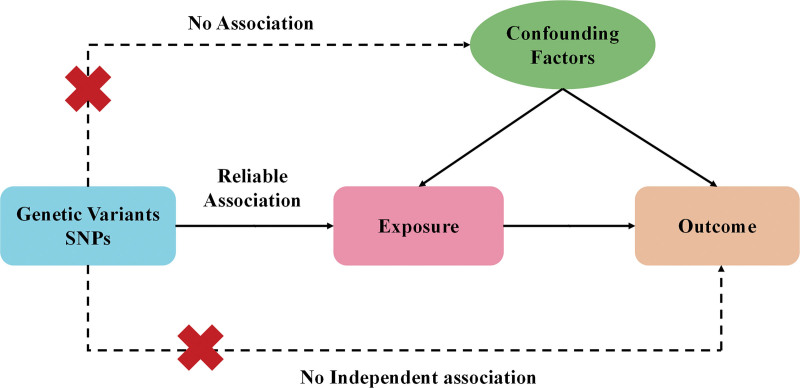
Three core assumptions of Mendelian randomization: relevance, independence, and exclusion restrictions.

Initially, we employed immune cell phenotypes to analyze which immune cell phenotypes may have potential causal relationships with the risk of HSIL. We subsequently used HSIL as exposures and explored the potential reverse causal relationships with immune cell phenotypes. Single nucleotide polymorphisms (SNPs) are utilized as instrumental variables (IVs) in this study. The selected IVs satisfy 3 crucial assumptions: IVs are associated with risk exposure. IVs are unrelated to any confounding factors influencing the exposure-outcome relationship. IVs can affect the outcome only through exposure and not through any other pathways. Any IVs violating the 3 major assumptions are excluded. The IVs selection criteria are shown in Figure 2.

We established the significance level for SNPs associated with immune cell phenotypes and HSIL at *P* < 1 × 10^−5^. A linkage disequilibrium (LD) assessment was performed on these SNPs, utilizing parameters of *r*² = 0.1 and kb = 500. The metrics *r*² or kb denote the extent of LD between 2 genetic loci, suggesting that when LD exists, allele frequencies at these loci are not independent but exhibit some correlation. We calculated the *F* statistic for each SNP, and SNPs with low *F* values (<10) were removed to mitigate weak instrument bias.

### 2.2. Data sources

Utilizing publicly accessible genetic information, we investigated the causal links between 731 immune cell signatures and the risk of HSIL. The summary statistics for immune traits were obtained from the genome-wide association study (GWAS) Catalog (https://www.ebi.ac.uk/gwas/), accession numbers from GCST0001391 to GCST0002121. A total of 731 immune cell phenotypes were included, encompassing absolute cell counts (n = 118), median fluorescence intensities indicating surface antigen levels (n = 389), morphological parameters (n = 32), and relative cell counts (n = 192).^[8]^ The traits of median fluorescence intensities, AC, and relative cell encompass a variety of immune cell types, such as B cells, CDCs, different stages of mature T cells, monocytes, myeloid cells, TBNK (which includes T cells, B cells, and natural killer cells), along with Treg panels. In contrast, the morphological parameters feature comprises CDC and TBNK panels. The original GWAS data was derived from a sample of 3757 individuals of European descent. Approximately 22 million SNPs were genotyped utilizing high-density arrays and were subsequently imputed using a Sardinian sequence-based reference panel.^[9]^ GWAS summary statistics for HSIL were obtained from Finngen R10 (https://r10.risteys.finngen.fi/). The study performed a GWAS on 2,30,310 European individuals (N_case_ = 6088, N_control_ = 2,24,222).

### 2.3. Statistical analysis

Data analyses were performed using R software (version 4.4.3). The primary method for MR analysis was inverse variance weighted (IVW). To evaluate the robustness of IVW, analyses utilizing weighted median, simple mode, MR-Egger, and weighted mode were conducted. Cochrane’s *Q* test was used to test for heterogeneity among SNPs, whereas the MR-Egger intercept was used to assess horizontal pleiotropy. Sensitivity was analyzed using the leave-one-out method, and outlier detection was carried out with Mendelian Randomization Pleiotropy RESidual Sum and Outlier. Scatter plots revealed that the results were not affected by outliers. Funnel plots demonstrated the robustness of the correlation and no heterogeneity. Comprehensive sensitivity analyses were used to verify the robustness, heterogeneity, and horizontal pleiotropy of the results. Given that the outcome indicators were binary variables, the results were reported as odds ratios and 95% CI. *P* < .05 was considered to indicate statistically significant differences.

To investigate reverse causality, identical approaches were applied for the reverse MR analysis concerning immune cell phenotypes and HSIL. Additionally, considering the issue of multiple testing, FDR correction was conducted. FDR < 0.20 is considered suggestive of a causal relationship, whereas FDR < 0.05 is considered to indicate a significant causal relationship.

## 3. Results

### 3.1. Exploration of the causal effect of immune cells on HSIL

**Figure 3.**
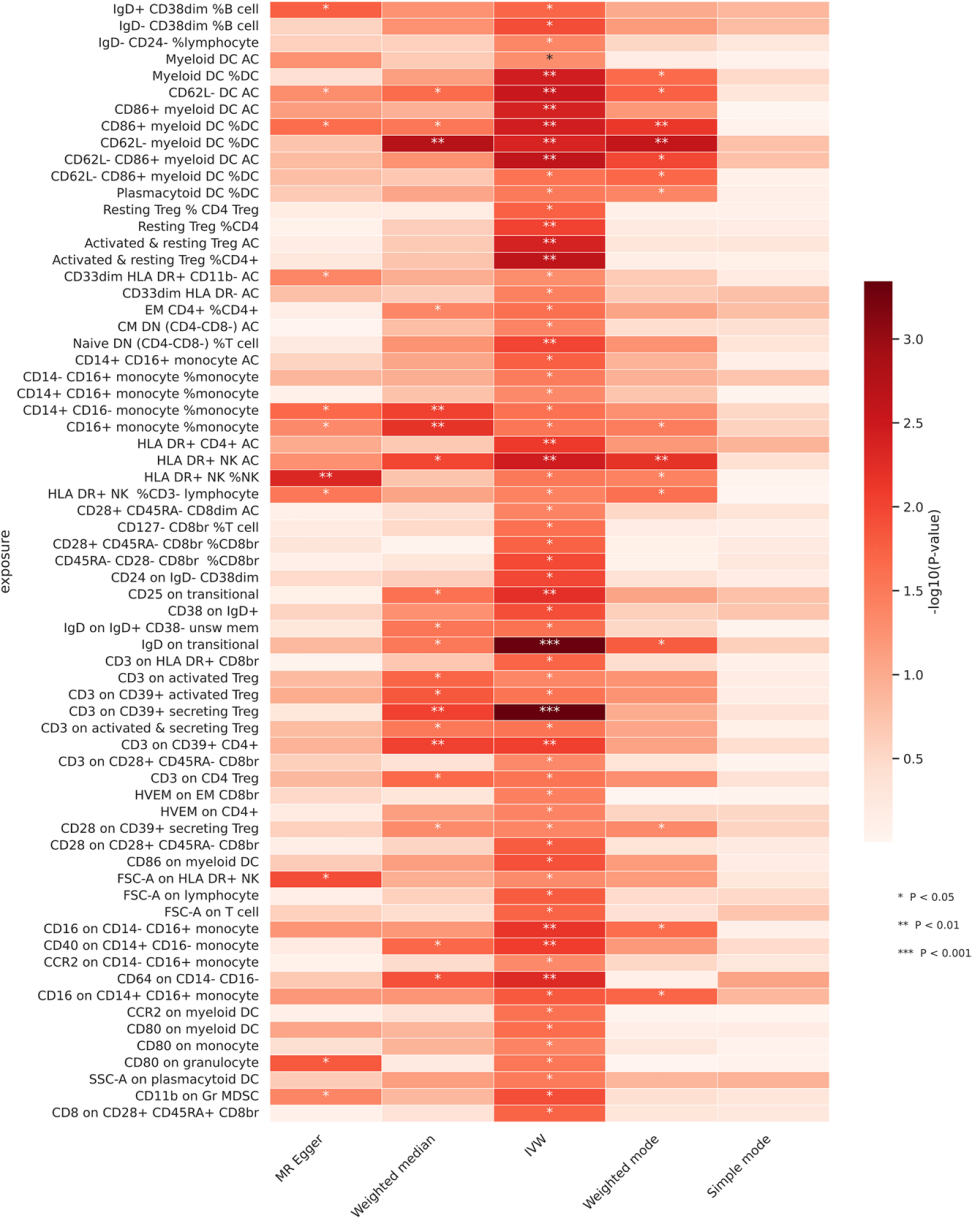
Heatmap of −log10 (*P*-values) for immune traits across 5 Mendelian randomization methods. *** *P* < .001; ** *P* < .01; * *P* < .05.

To explore the causal effects of immune cell phenotypes on HSIL, 2-sample MR analysis was performed, and the IVW method was used for the main analysis. At the 0.05 significance level, a total of 67 immune cell phenotypes were identified as causally associated with the development of HSIL. The results of the analysis are shown in Figure 3.

**Figure 4.**
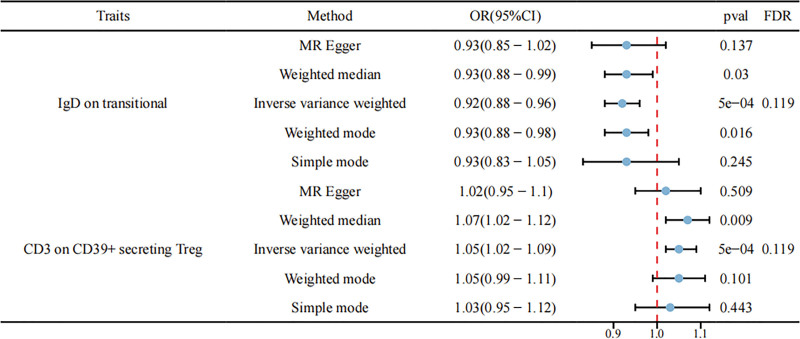
Forest plots showing the causal effect of immune cell phenotypes on HSIL using 5 methods: IgD on transitional was significantly negatively correlated with the risk of HSIL whereas CD3 on CD39+ secreting Treg was significantly positively correlated. CI = confidence interval, HSIL = high-grade squamous intraepithelial lesion, OR = odds ratio.

After multiple test adjustments via the FDR method (FDR < 0.05), no immune cell phenotypes were identified at a significance level of 0.05. However, at FDR < 0.20, 2 suggestive immune cell phenotypes were identified. Specifically, IgD on transitional (OR = 0.92, 95% CI: 0.88–0.96, *P* = .0005, FDR = 0.119) was significantly negatively correlated with the risk of HSIL, whereas CD3 on CD39+ secreting Treg (OR = 1.05, 95% CI: 1.02–1.09, *P* = .0005, FDR = 0.119) was significantly positively correlated with the risk of HSIL. These results can be found in Figure 4. A horizontal pleiotropy test was subsequently conducted using MR Egger and Mendelian Randomization Pleiotropy RESidual Sum and Outlier in combination. No horizontal pleiotropy was detected in the above results, and Cochran’s *Q* test revealed no heterogeneity in any of the outcomes (Supplementary 1, Supplemental Digital Content, https://links.lww.com/MD/Q595). Scatter plots and funnel plots also supported these findings (Supplementary 2, Supplemental Digital Content, https://links.lww.com/MD/Q595).

### 3.2. The causal effect of HSIL on immune cells

**Figure 5.**
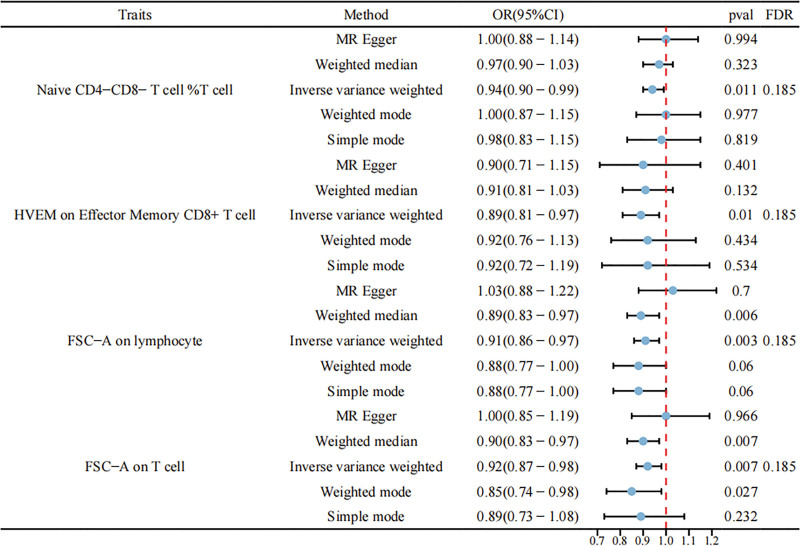
Forest plots showing the causal effect of HSIL on 4 immune cell phenotypes. CI = confidence interval, HSIL = high-grade squamous intraepithelial lesion, OR = odds ratio.

In the reverse MR analysis, we identified some positive results, but after FDR correction (FDR < 0.05), no statistically significant results were observed. After adjusting to FDR < 0.20, there were 4 immune cell phenotypes, which included naive CD4− CD8− T cell %T cell (OR = 0.94, 95% CI: 0.90–0.99, *P* = .011, FDR = 0.185), HVEM on effector memory CD8+ T cell (OR = 0.89, 95% CI: 0.81–0.97, *P* = .010, FDR = 0.185), FSC-A on lymphocyte (OR = 0.91, 95% CI: 0.81–0.97, *P* = .010, FDR = 0.185), and FSC-A on T cells (OR = 0.92, 95% CI: 0.87–0.98, *P* = .007, FDR = 0.185). These results can be found in Figure 5. Given the presence of horizontal pleiotropy and heterogeneity in the reverse MR analysis (Supplementary 3, Supplemental Digital Content, https://links.lww.com/MD/Q595), the causal inference from HSIL to immune phenotypes should be interpreted with caution.

## 4. Discussion

Through a 2-sample bidirectional MR study, we identified 67 immune cell phenotypes significantly associated with the risk of HSIL (*P* < .05). After adjusting for FDR < 0.20, 2 immune cell phenotypes were found to be related to the risk of HSIL. In the reverse MR analysis, we also observed some positive results; however, after FDR correction (FDR < 0.20), 4 immune cell phenotypes were identified.

To ensure the robustness of our findings and mitigate the potential influence of horizontal pleiotropy or invalid instruments, we employed 5 different MR methods: IVW, weighted median, MR-Egger, simple mode, and weighted mode. While IVW served as our primary analytical approach due to its superior statistical power under the assumption that all instruments are valid, the additional methods offer complementary strengths under less stringent assumptions. The direction and magnitude of the causal estimates were broadly consistent across methods, though significance levels varied. Notably, IVW yielded statistically significant results for both traits, while MR-Egger, weighted median and simple mode produced estimates in the same direction but with reduced significance, likely due to reduced statistical power and a limited number of instruments.

Our MR analysis revealed a suggestive protective association between IgD expression on transitional B cells and HSIL risk (OR = 0.92, 95% CI: 0.88–0.96, *P* = .0005), although the effect did not survive multiple testing correction (FDR = 0.119). This finding aligns with emerging evidence on the immunomodulatory role of IgD in mucosal immunity and B cell biology.^[10]^ Transitional B cells, positioned at the interface of immature and mature B cell pools, undergo IgD class-switching recombination in mucosal lymphoid tissues, a process dependent on activation-induced cytidine deaminase and regulated by T-cell-dependent and T-cell-independent pathways.^[11]^ Notably, mucosal IgD+ B cells exhibit unique functional plasticity, secreting IgD antibodies that bind commensal and pathogenic antigens while interacting with innate immune cells such as basophils and mast cells via galectin-9/CD44 receptors.^[12,13]^ This dual capacity for antigen recognition and innate immune activation may underpin the potential protective effects of IgD against HSIL, possibly by enhancing local antimicrobial responses or dampening HPV-driven inflammation.

MR analysis also revealed a significant positive association between CD3 expression on CD39+ secreting Treg and the risk of HSIL (OR = 1.05, 95% CI: 1.02–1.09, *P* = .004). This finding aligns with emerging evidence indicating that CD39+ Tregs are involved in immune dysregulation during precancerous progression. CD39 (ENTPD1) is a cell-surface ectonucleotidases that contributes to regulate TIME and immunotherapy response through the hydrolysis of extracellular ATP and ADP, leading to immunosuppressant adenosine generation.^[14–16]^ CD39+ Tregs enhance immunosuppressive activity by promoting immune evasion in malignancies such as non-small cell lung cancer and colon adenocarcinoma.^[17]^ The observed association between CD3 (T cell co-receptor) expression and HSIL risk suggests that TCR activation in CD39+ Tregs may amplify their adenosine-mediated immunosuppressive capacity. Specifically, CD3 engagement can stabilize Foxp3 expression and upregulate inhibitory cytokines such as TGF-β, fostering a tolerogenic microenvironment conducive to persistent HPV infection and cervical dysplasia.^[18]^ In addition, HSIL may involve localized CD39 upregulation in the cervical epithelium, driven by chronic inflammation or HPV oncoprotein E6/E7-mediated metabolic reprogramming.^[19,20]^

These findings, while biologically plausible, are based on a suggestive significance threshold (FDR < 0.20), and should be interpreted with caution. Given the liberal threshold, the associations are best considered exploratory and hypothesis-generating. Further validation in larger, independent cohorts and experimental models will be necessary to confirm these potential causal relationships.

Our reverse MR analysis revealed suggestive evidence (FDR < 0.2) of causal associations between HSIL and immune cell phenotypes, providing novel insights into the immunomodulatory effects of precancerous cervical lesions. First, the inverse association between the HSIL and naïve CD4−CD8− T cell %T cell (OR = 0.94, *P* = .011) aligns with the findings of single-cell studies showing reduced naïve T-cell proportions in HPV-infected cervical lesions, likely reflecting chronic antigen exposure and immune exhaustion.^[21]^ Depletion of naïve T cells may compromise adaptive immunity, facilitating persistent HPV infection and progression to ICC.^[22]^ CD4−CD8− T cells may represent a mixture of unconventional and non-MHC-restricted T cell subsets, including γδ T cells, NKT cells, and MAIT cells, all of which are enriched at mucosal barriers and contribute to antiviral and antitumor immunity.^[23–26]^ Therefore, the observed associations between HSIL and double-negative T cells may reflect the involvement of these specialized populations in cervical immune surveillance and carcinogenic progression. Second, the association between HSIL and HVEM on effector memory CD8+ T cells (OR = 0.89, *P* = .010) suggests that HSIL disrupts HVEM-mediated coinhibitory signaling, a pathway critical for balancing T-cell activation and tolerance.^[27]^ Downregulation of HVEM on effector memory CD8+ T cells could impair their cytotoxic function and promote immune evasion, as observed in tumor microenvironments characterized by CD8+ T-cell exhaustion.^[28]^ Third, reduced FSC-A on lymphocytes (OR = 0.91, *P* = .010) and FSC-A on T cells (OR = 0.92, *P* = .007) indicate that HSIL may induce cellular quiescence, as FSC-A reflects cell size and activation status. A smaller lymphocyte size has been linked to diminished effector function, potentially creating an immunosuppressive niche favorable for HSIL persistence.^[29,30]^ While these associations did not survive strict FDR correction, their biological plausibility and consistency with prior mechanistic studies suggest that HSIL causally alters immune surveillance pathways. Future studies integrating single-cell profiling and functional validation are warranted to confirm these relationships and explore therapeutic strategies targeting T-cell exhaustion or HVEM signaling in cervical precancerous lesions.

Our MR analyses were based on immune cell phenotypes derived from peripheral blood, which may not fully reflect the immune microenvironment within the cervical epithelium. While peripheral blood provides a practical and widely available source for large-scale genetic studies, it does not capture the spatial organization, activation states, or tissue-specific signals of immune cells at mucosal sites of the cervix. Nonetheless, prior studies have shown that systemic immune alterations can correlate with local immune dysregulation in the context of persistent viral infections, including HPV.^[21,31,32]^ For instance, T-cell exhaustion markers and regulatory cell subsets in blood have been associated with cervical disease severity.^[33,34]^ Therefore, although peripheral immune traits provide valuable insights, our findings should be interpreted with caution and validated in tissue-level studies, such as cervical single-cell or spatial transcriptomic analyses.^[35]^

The strengths of our study include the first application of MR methods to investigate the relationship between immune cell phenotypes and HSIL. Our conclusions were derived under strict examination of horizontal pleiotropy, reducing the interference of confounding factors and the impact of reverse causality on the results. Additionally, our study revealed that immune cell phenotypes are significantly associated with HSIL, which has been less explored in previous studies. These findings may provide new insights for exploring potential immunotherapeutic targets in HSIL. Our study also has several limitations. Although we included 731 immune cell phenotypes in our research, some immune cell phenotypes could not be analyzed due to data limitations. Additionally, since the data sources are predominantly of European descent, limited to adults, and do not support stratification by sex and age, this may impact the generalizability and accuracy of the results. This study is based on a cohort study of individuals from Sardinia. The Sardinian population possesses unique genetic characteristics, and the conclusions of this study may not be applicable to broader populations. Future validation in larger and more diverse patient cohorts is necessary to ensure the robustness and generalizability of our conclusions. Furthermore, we acknowledge that the use of a relatively high FDR threshold (0.20) increases the risk of false positives. As such, these results should be considered preliminary and require validation through future replication studies. Finally, we hope that future research will involve larger sample sizes and more comprehensive MR studies to further explore the relationship between immune cell phenotypes and HSIL.

## 5. Conclusions

This bidirectional 2-sample MR study provides genetic evidence supporting the causal role of immune cell phenotypes in the pathogenesis of HSIL. This work provides a mechanistic framework for HSIL pathogenesis and charts a roadmap for precision immunotherapy, offering transformative potential for early intervention and personalized management of cervical precancerous lesions. However, future research using independent datasets and functional validation will be essential to confirm their translational potential.

## Acknowledgments

We thank the GWAS database contributors for sharing the data.

## Author contributions

**Conceptualization:** Zhenyu Wang, Ping Jiang, Junjie Wang.

**Data curation:** Lu Yang.

**Formal analysis:** Zhenyu Wang.

**Investigation:** Lu Yang, Junjie Wang.

**Methodology:** Zhenyu Wang.

**Supervision:** Lu Yang, Chunxiao Li, Ping Jiang, Junjie Wang.

**Writing – original draft:** Zhenyu Wang.

**Writing – review & editing:** Ping Jiang, Junjie Wang.

Supplemental Digital Content “Supplementary 4, 5 & 6” are available for this article (https://links.lww.com/MD/Q596).

## Supplementary Material




