# Supplementary material for: A bidirectional Mendelian randomization analysis of the causal relationship between immune cells and high-grade squamous intraepithelial lesion
Source: Medicine (Baltimore). 2025 Nov 21;104(47):e45695. doi: 10.1097/MD.0000000000045695 (PMC12643692; doi:10.1097/MD.0000000000045695)
Supplement: Supplementary file 1 [file medi-104-e45695-s001.pdf]

Table 1 The results of the heterogeneity (Cochran's Q test)

Trait	Cochran's Q	df	P-value
IgD on transitional	57.89690263	41	0.061885509
CD3 on CD39+ secreting Treg	63.84230808	58	0.278709759

We conducted Cochran's Q test to assess heterogeneity across instrumental variables. The P-values for all primary analyses were > 0.05, indicating no significant heterogeneity among SNPs. Therefore, the IVW estimates are considered robust and consistent across instruments. The Q statistics and P-values are now reported in Table 1.

Table 2 Horizontal pleiotropy (MR-Egger intercept)

Trait	MR-Egger Intercept	SE	P-value
IgD on transitional	-0.002236062	0.010167106	0.827043484
CD3 on CD39+ secreting Treg	0.008194464	0.009255139	0.379663018

Table 3 Horizontal pleiotropy (MRPRESSO)

Trait	Global Test P	Outliers Detected
IgD on transitional	0.312	NA
CD3 on CD39+ secreting Treg	0.292	NA

We have included MR-Egger intercept and MR-PRESSO results in Table 2 and Table 3 . All tested traits showed P > 0.05 for both the MR-Egger intercept and MR-PRESSO global test, indicating no significant directional pleiotropy or outlier SNPs influencing the results. Therefore, the IVW causal estimates are considered robust.

Supplementary 2 All plots were generated using the TwoSampleMR and MR-PRESSO packages in R.

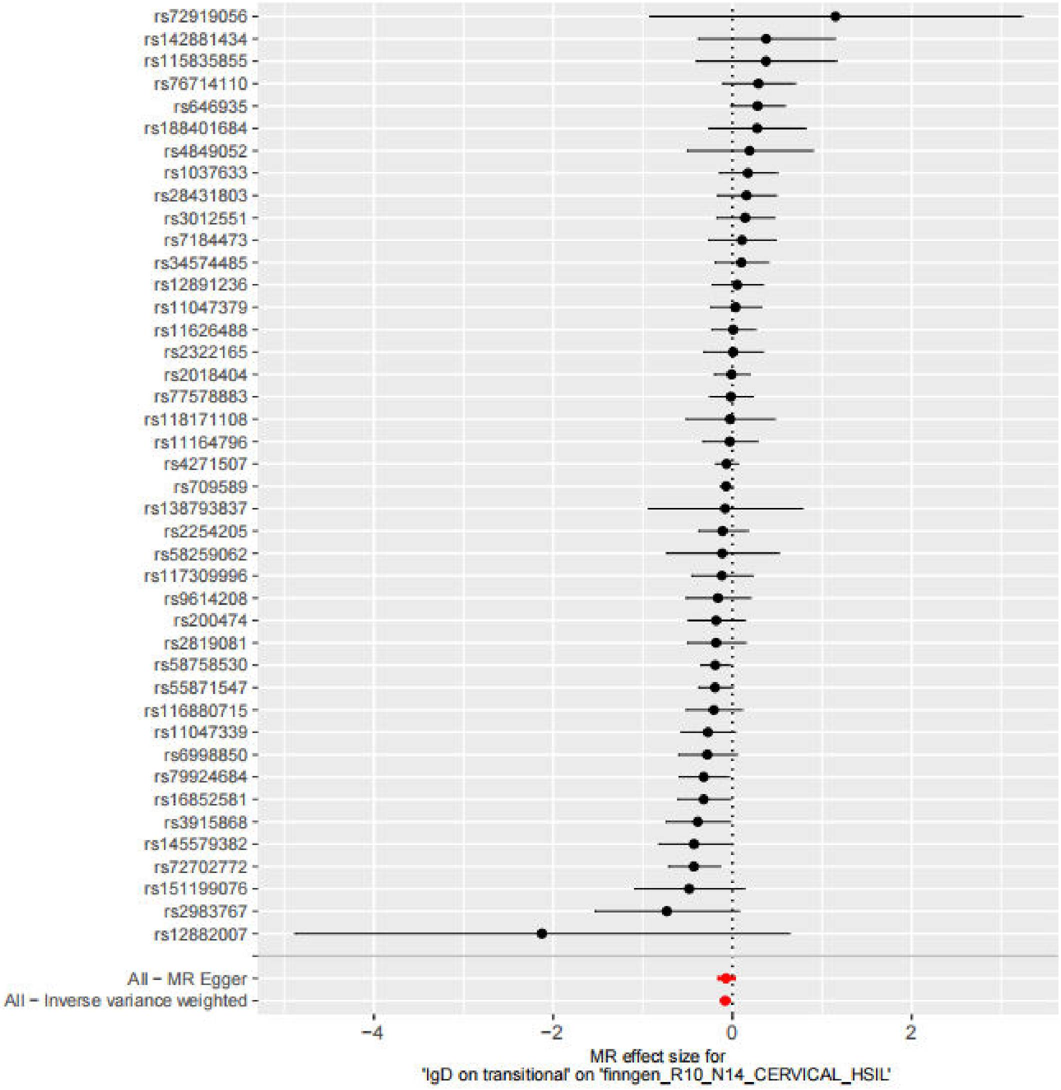

Figure 1 Forest plot showing the causal effect estimates for individual SNPs for IgD on transitional.

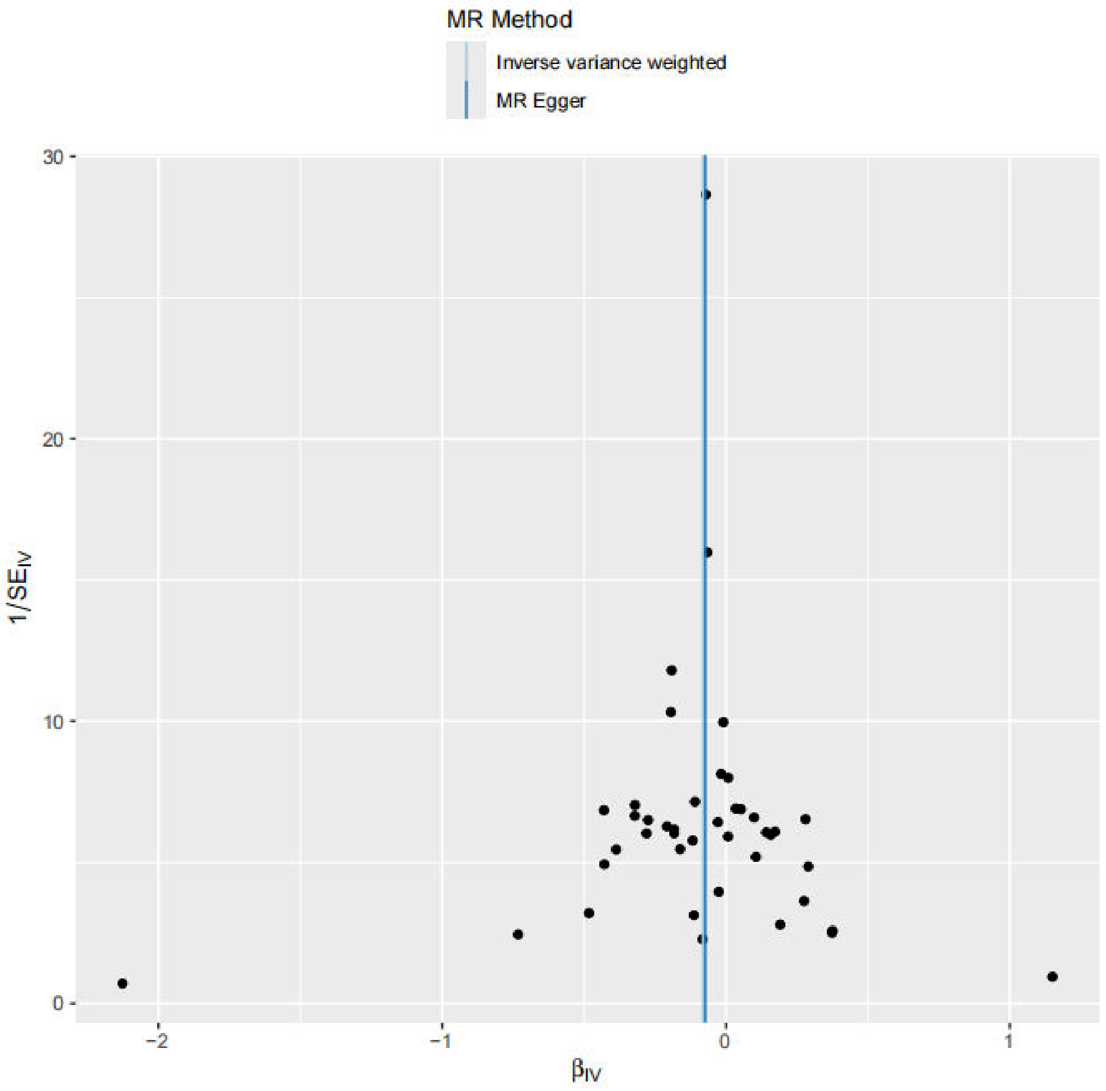

Figure 2 Funnel plots showing the distribution of the effect estimates for individual SNPs. The symmetry of the plots suggests the absence of directional horizontal pleiotropy.

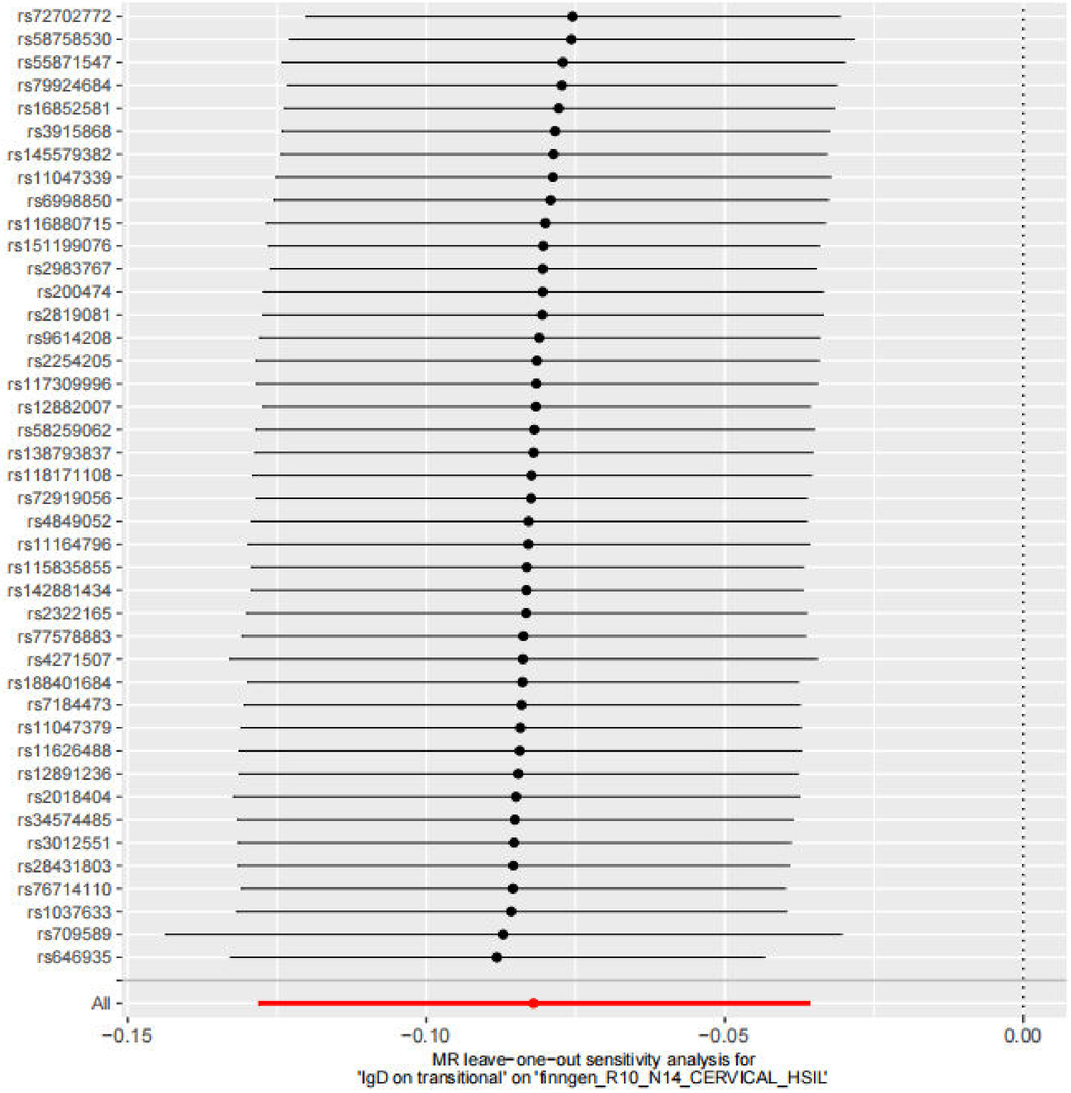

Figure 3 Leave-one-out sensitivity analysis of MR estimates. Each point represents the overall causal estimate recalculated after removing one SNP at a time. The consistency of estimates across iterations indicates that the results are not driven by any single instrumental variable.

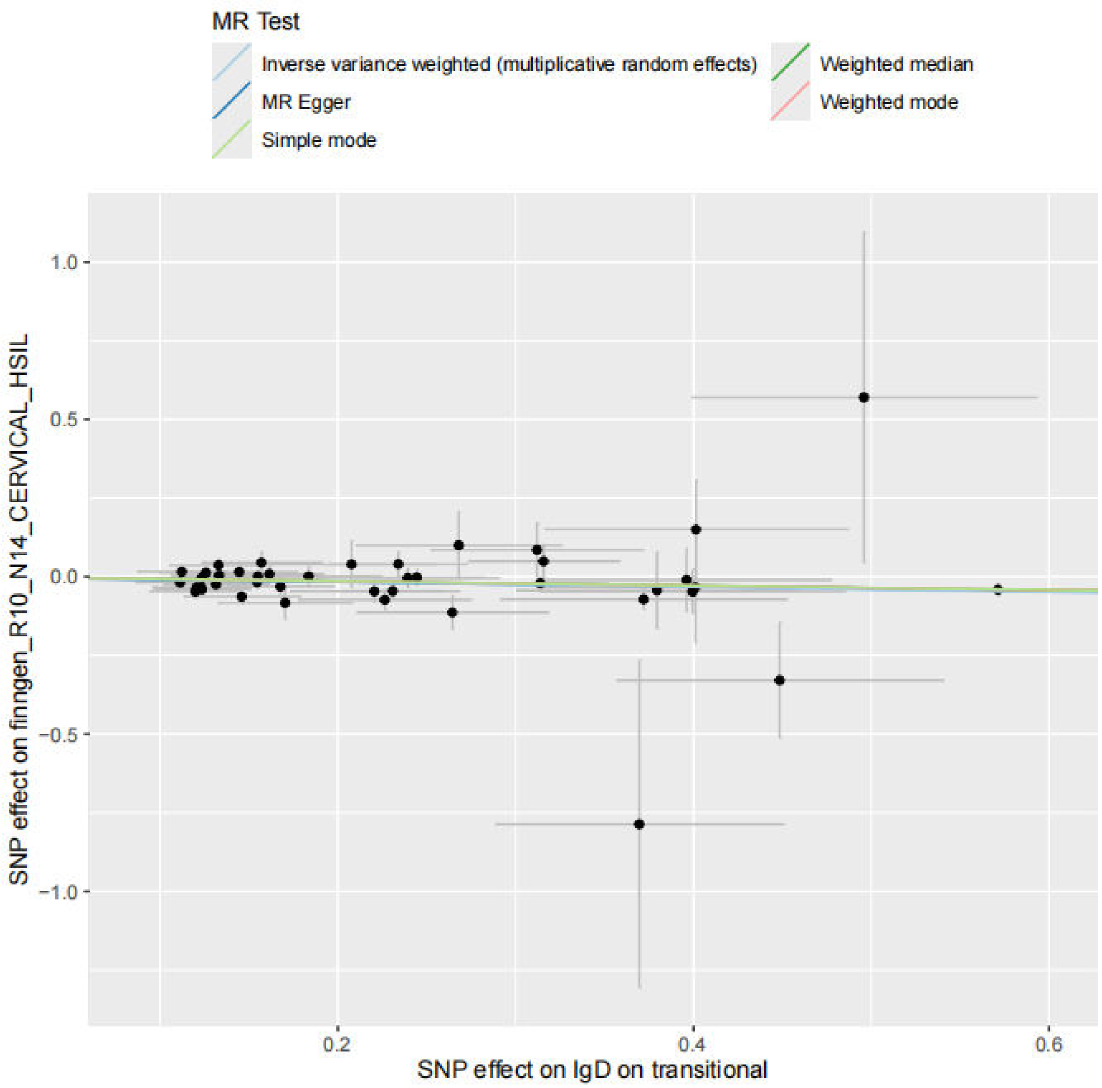

Figure 4 Scatter plots showing causal estimates of IgD on transitional on HSIL using various MR methods.

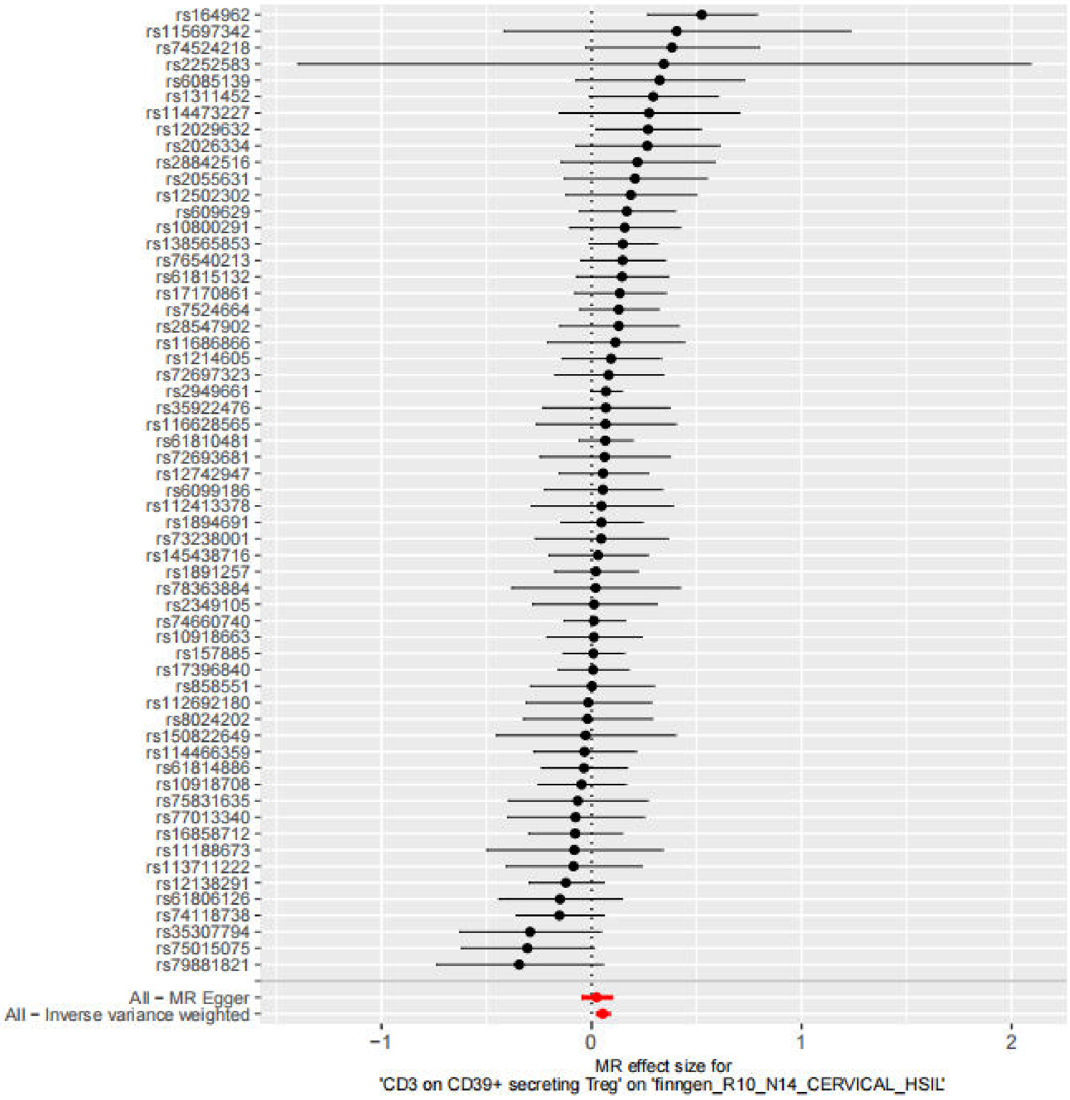

Figure 5 Forest plot showing the causal effect estimates for individual SNPs for CD3 on CD39+ secreting Treg.

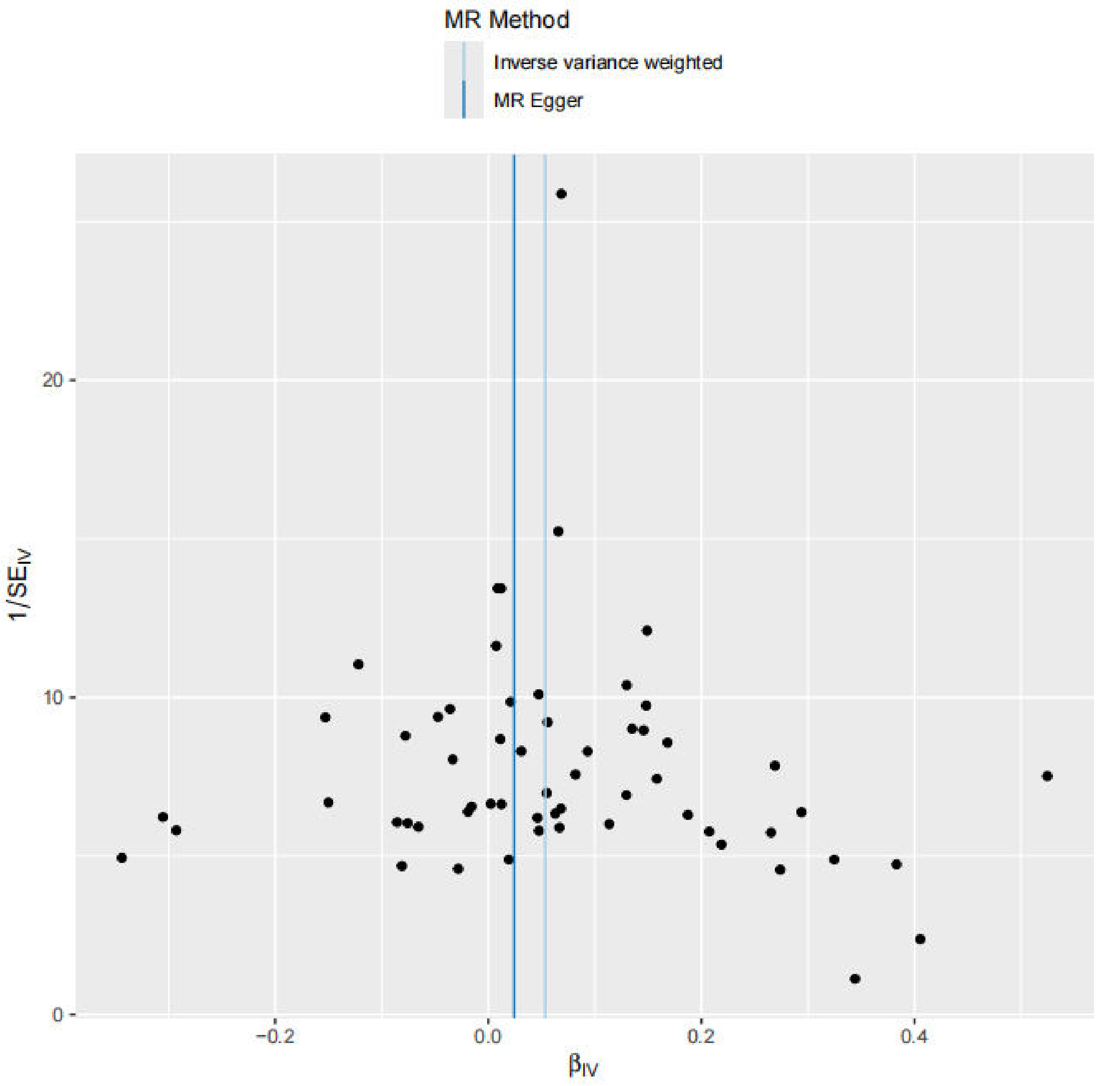

Figure 6 Funnel plots showing the distribution of the effect estimates for individual SNPs. The symmetry of the plots suggests the absence of directional horizontal pleiotropy.

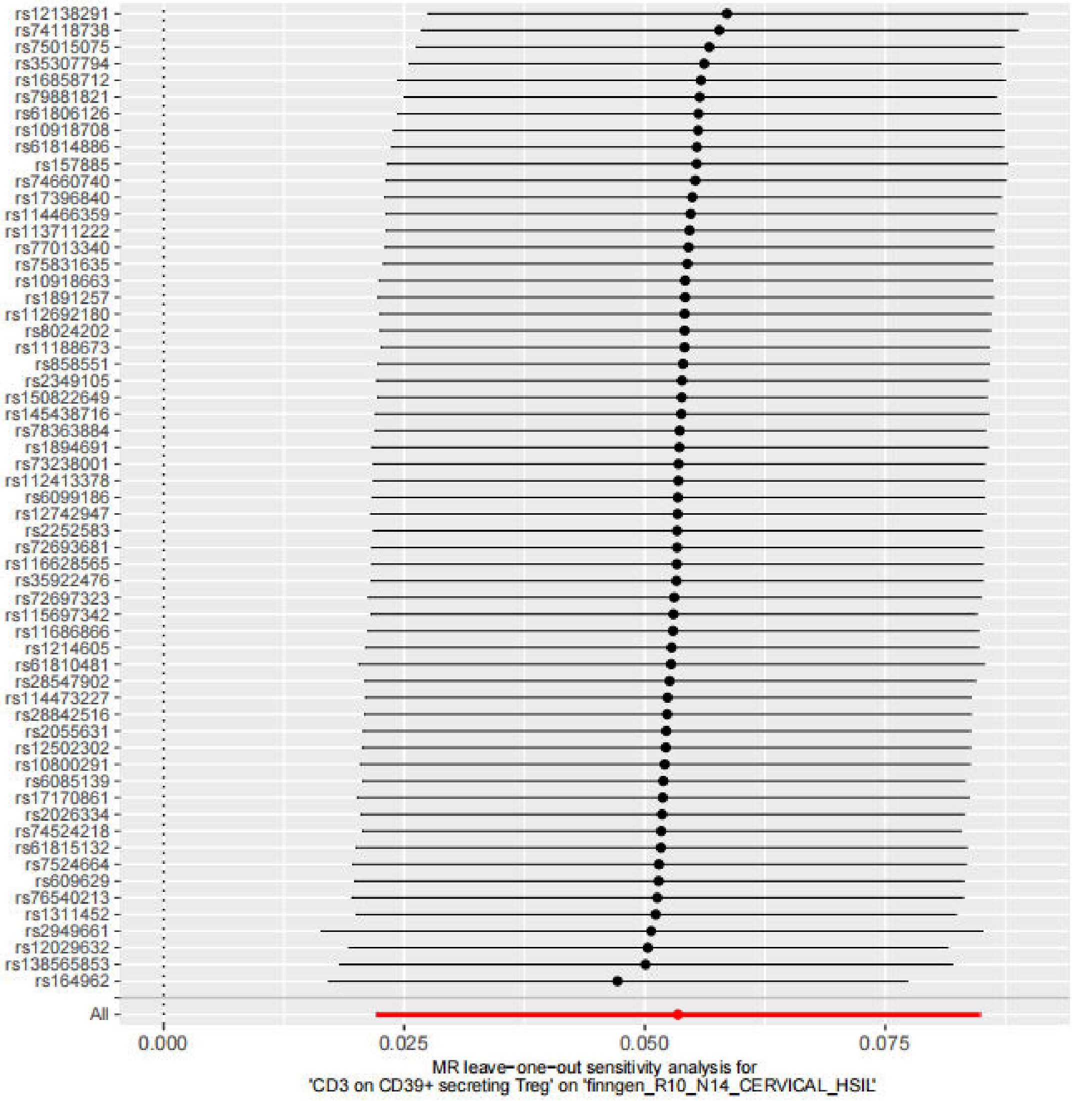

Figure 7 Leave-one-out sensitivity analysis of MR estimates. Each point represents the overall causal estimate recalculated after removing one SNP at a time. The consistency of estimates across iterations indicates that the results are not driven by any single instrumental variable.

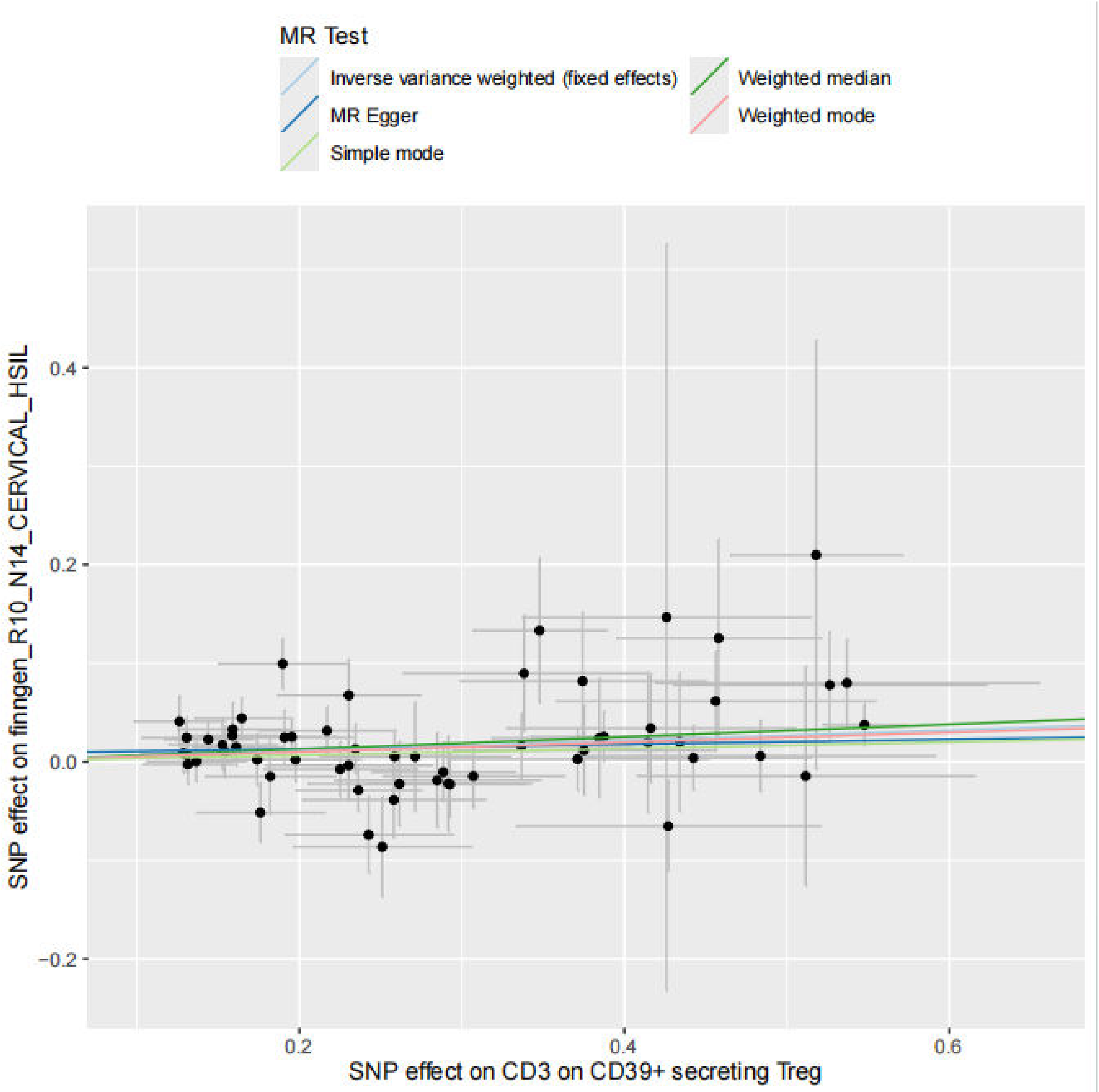

Figure 8 Scatter plots showing causal estimates of CD3 on CD39+ secreting Treg on HSIL using various MR methods.

Table 1 The results of the heterogeneity (Cochran's Q test)

Trait	Cochran's Q	df	P-value
Naive CD4-CD8- T cell %T cell	123.938758	99	0.045645402
HVEM on Effector Memory CD8+ T cell	128.2773604	100	0.029834547
FSC-A on lymphocyte	152.5426205	100	0.000562763
FSC-A on T cell	153.5846451	100	0.000461779

Table 2 Horizontal pleiotropy (MR-Egger intercept)

Trait	MR-Egger Intercept	SE	P-value
Naive CD4-CD8- T cell %T cell	-0.00966013	0.009381234	0.305671898
HVEM on Effector Memory CD8+ T cell	-0.002269966	0.017887134	0.89927302
FSC-A on lymphocyte	-0.018891147	0.011980943	0.118039517
FSC-A on T cell	-0.013419131	0.01235712	0.280141229

Table 3 Horizontal pleiotropy (MRPRESSO)

Trait	Global Test P	Outliers Detected
Naive CD4-CD8- T cell %T cell	0.002	rs2239701, rs62017981, rs71521005, rs9277196, NA
HVEM on Effector Memory CD8+ T cell	0.002	rs11756541, rs1963304, rs2844494, NA
FSC-A on lymphocyte	0.002	rs113547322, rs1432286, rs2844494, rs9276606
FSC-A on T cell	0.002	rs113547322, rs1432286, rs2844494, rs72865631
